# Some Notes on Phthisis—I

**Published:** 1908-11-28

**Authors:** 


### SOME NOTES ON PHTHISIS—I.

### TOBACCO-SMOKING AND TUBERCULOSIS.

The Henry Phipps Institute for the Study, Treatment, and Prevention of Tuberculosis makes a most thorough annual report, and it is interesting to note some of the points from its fourth issue.

Accurate statistics have been drawn up on the relations between the use of tobacco and the incidence of pulmonary tuberculosis. The figures are so far available only for one year, but they are already instructive.

No women are recorded as having used tobacco, so that they are excluded from consideration altogether; that is to say, the issue is not confused by the inclusion of the women among the non-smokers. Altogether there were 443 male patients during the year, and of these 322 used tobacco in one form or another; 119 did not use tobacco at all, and in the remaining two no record was made upon the question. Of the users of tobacco 199 smoked only, 27 chewed only, and 96 both smoked and chewed. The results were as follows:—

Out of 322 Who Gave a History of Use of Tobacco,

2 were discharged with the disease arrested.
101 were discharged improved.
116 were discharged unimproved.
50 died.
53 made only one visit.

Out of 119 Who Gave a History of not Being Users of Tobacco,

2 were discharged with the disease arrested.
44 were discharged improved.
46 were discharged unimproved.
5 died.
22 made only one visit.

Expressed in another way, the disease was improved or arrested in 38 per cent. of those who used tobacco and in 47 per cent. of those who did not use it, while 62 per cent. of the tobacco users did unfavourably against 53 per cent. of the others. The preponderance seems sufficient to disprove the claim which has been made that tobacco is a preventive of tuberculous implantation.

It is to be hoped that the above statistics will be continued and elaborated as years go on. One would like to know the relative merits and demerits, as regards phthisis, of cigarettes, cigars, and pipes respectively; of each of these as regards amounts per week, and in relation to indoor or outdoor occupations; of the variations in phthisis incidence that may occur among those who do and those who do not inhale; those who chew and those who do not chew; those who snuff and those who do not.

One would like, for example, to know with some degree of certainty whether pipe smokers who inhale or cigar smokers who inhale do worse than those who only inhale the smoke of cigarettes; whether a smoker who contracts phthisis and is still in an early stage of the disease is more likely to recover if he continues smoking as before, reduces his smoking, or gives it up altogether. It is surprising how difficult it is to obtain a definite answer to these questions beyond the personal opinion of individuals. Such personal opinion is given by some in favour of continuance of moderate smoking, by others in favour of relinquishing it. There are no real data for either opinion. The data are only now being accumulated. They are open to the very greatest difficulties in interpretation owing to the wide differences there are in other factors in the different cases. The future reports upon the question from the Phipps Institute cannot but be looked forward to with interest.

